# An Interesting Case of a Cold Left Arm

**DOI:** 10.7759/cureus.11524

**Published:** 2020-11-17

**Authors:** Laura Hmiel, Attila Nemeth

**Affiliations:** 1 Internal Medicine, University Hospitals Cleveland Medical Center, Cleveland, USA; 2 Internal Medicine, Louis Stokes Cleveland VA Medical Center/Case Western Reserve University School of Medicine, Cleveland, USA

**Keywords:** coronary artery bypass graft surgery, subclavian artery thrombosis, lima, atherosclerosis, ventricular tachycardia (vt), non valvular atrial fibrillation

## Abstract

Inter-arm variability in blood pressure readings typically signifies arterial disease between the aortic arch and the subclavian artery. The differential diagnosis includes thoracic aortic dissection, atherosclerosis, thoracic outlet syndrome, and subclavian artery stenosis and thrombosis. In patients with prior coronary artery bypass grafting, including the internal mammary artery, several of those conditions can compromise coronary blood flow and lead to myocardial ischemia. Here we discuss a case of left subclavian artery thrombosis, which compromised left internal mammary artery blood flow and led to ischemic ventricular tachycardia.

## Introduction

Symptomatic subclavian artery thrombosis is a rare condition which is often associated with and caused by atherosclerosis and can ultimately lead to critical limb ischemia if not recognized promptly [1]. Unfortunately, many of the presenting symptoms and signs of this condition, including inter-arm variability in blood pressure, can be present in several other conditions associated with atherosclerosis which require very different treatments with varying levels of urgency. Thoracic outlet syndrome, characterized by mechanical compression of the neurovascular bundle as it leaves the thoracic cavity through the thoracic outlet, leads to pain, pallor, and inter-arm variability in blood pressure when the subclavian artery is affected [2]. Subclavian artery steal syndrome also presents with ischemic symptoms of pain and pallor. It can occur when subclavian artery atherosclerosis causes alterations in blood flow and pressures on either side of the stenosis [3]. Emergent and potentially life- or limb-threatening conditions include thoracic aortic dissection [4] as well as subclavian artery thrombosis [1]. Given the high prevalence of the atherosclerotic disease, including cardiac and peripheral atherosclerosis, subclavian artery thrombosis should be considered in any patient presenting with characteristic signs and symptoms, including arm pain, paresthesia, and pulselessness.

## Case presentation

**Figure 1:**
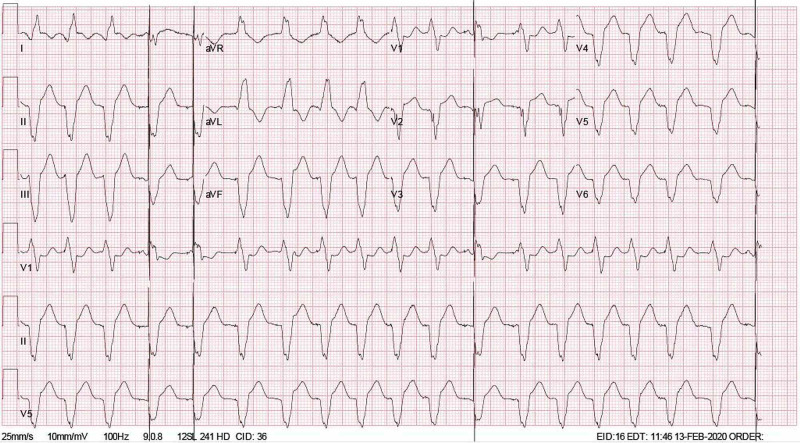
Electrocardiogram on initial presentation showing CRT pacing. CRT- Cardiac resynchronization therapy

The patient was a 72-year-old man with a history of coronary atherosclerosis and anterior myocardial infarction who underwent a coronary artery bypass graft in 2001. He had in total three grafts: left internal mammary artery (LIMA) to left anterior descending artery, saphenous vein to the right coronary artery, and saphenous vein to ramus intermedius. He presented to the emergency department in February of 2020 with a three-day history of left shoulder pain and a one-day history of left arm tingling. His vital signs at triage were notable for a pulse of 94 beats per minute and blood pressure of 132/94 mmHg. His ECG demonstrated a paced ventricular rhythm at a rate of 111 beats per minute (Figure 1). He denied any associated chest pain or neurologic symptoms.

His past medical history included Hurthle cell cancer of the thyroid, which was treated with a thyroidectomy in 2015, followed by radiation to the thyroid bed, neck, and mediastinal nodes in 2017. His history also included ischemic cardiomyopathy with anterior and apical wall motion abnormalities, left ventricular ejection fraction of 20-25%, and ventricular tachycardia (VT) storm, for which a cardiac resynchronization therapy defibrillator (CRT-D) was placed for secondary prevention. Regarding his defibrillator, he previously experienced a lead fracture and lead replacement. He subsequently developed a left ventricular (LV) thrombus, found on echocardiogram 11 months before presentation, which was treated with warfarin. A repeat echocardiogram three months before presentation showed resolution of the LV thrombus. His cardiologist advised him to stop warfarin at this time. He was maintained on dofetilide for VT storm prevention.

Eleven days before presentation, he had a run of VT which was successfully terminated with one round of anti-tachycardia pacing by his CRT-D. His electrophysiology device nurse subsequently reviewed his defibrillator and also noted a 9.2% burden of atrial tachycardia-atrial fibrillation (AT-AF). These episodes were attributed to a flu-like illness the patient was experiencing, and a note was made in the patient chart to discuss restarting anticoagulation with the patient given his new AT-AF.

**Figure 2:**
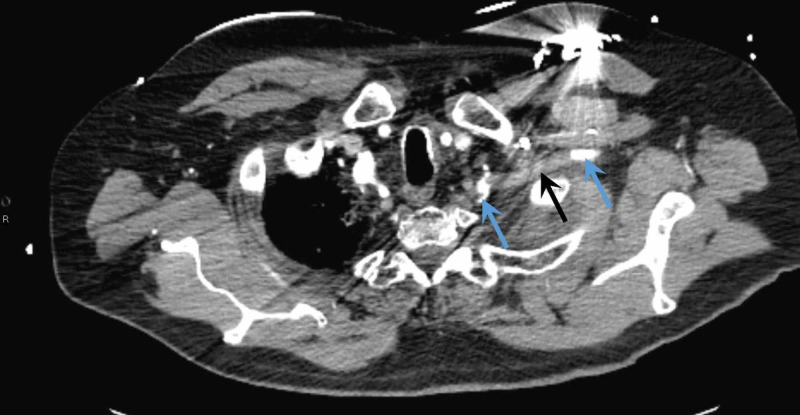
Transverse CT image with arterial phase contrast showing patent portions of the left subclavian artery (blue arrows) and the occlusion (black arrow).

When roomed in the emergency department, his systolic blood pressure taken in his left arm decreased to approximately 95/60 mmHg without any change in consciousness or symptomatology; the blood pressure in his right arm was similar to his initial measurement at triage. His left radial pulse was absent. Soon after this discovery, his pulse rose to 170-180 beats per minute, which prompted his CRT-D to provide a shock. Pacemaker interrogation confirmed VT. He was sent for emergent CT angiography to assess for aortic dissection due to the discrepancy in the upper extremity blood pressures. The CT (Figure 2) demonstrated an occluded mid-left subclavian artery without opacification of the left vertebral artery. The LIMA was not well visualized.

Aortic dissection or aneurysm was not observed. He was admitted to the cardiology intensive care unit with vascular surgery consultation for acute limb ischemia. He was started on a heparin drip for subclavian arterial thrombosis causing myocardial ischemia and started on lidocaine for ischemia-mediated VT; notably, his troponin rose from 0 ng/ml to 1.78 ng/ml (normal <0.05 ng/ml) five hours after presentation. Coronary catheterization noted chronic total occlusion of the left anterior descending artery with collaterals, a patent right coronary artery vein graft, and proximal occlusion of the left subclavian artery with an inability to visualize or engage the LIMA. Runoff of contrast into the left arm demonstrated low flow in the radial artery, but there were collateral arteries from other vessels, suggesting a chronic occlusion. No intervention was performed during the catheterization. After admission, the medical team discussed the goals of care with the patient. He wished to avoid further surgical or procedural interventions. He enrolled in hospice, and his CRT-D was shut off. Later that day, he expired from VT cardiac arrest noted on telemetry. 

## Discussion

This patient's case illustrated the diagnostic lesson that discordant upper extremity blood pressures with unilateral paresthesia have a critical differential diagnosis. Often in emergency settings, the clinician considers aortic dissection to be the biggest "can't miss diagnosis" on the differential, as the mortality of an acute aortic dissection is incredibly high, particularly with ascending thoracic aortic dissections. For such aortic dissections, the mortality rate increases by 1-2% every hour after symptom onset without surgical intervention [5]. With medical therapy alone, ascending thoracic aortic dissection has a mortality rate of at least 50% [4]. However, any arterial insult or pathology affecting the large arteries in the upper extremities can also present with a blood pressure difference of greater than 20mmHg. These other essential differential diagnoses include subclavian artery steal syndrome related to arterial stenosis and arterial thrombosis.

Coronary-subclavian steal syndrome is well documented in the literature [3] and occurs in patients with both subclavian artery stenosis and prior coronary artery bypass graft. Coronary-subclavian steal syndrome is a result of ostial or proximal subclavian artery stenosis which leads to reversal of blood flow from the grafted coronary artery in order to supply the more distal subclavian artery, particularly when that arm is moving and has increased blood and oxygen demand. This steal phenomenon can result in angina or myocardial infarction due to the temporary reversal in blood flow from the coronary artery. A similar phenomenon can occur with vertebrobasilar-subclavian steal syndrome, where blood flow is "stolen" from the brain, and neurologic symptoms predominate [6]. Subclavian artery stenosis itself is estimated to occur in up to 54% of those with peripheral vascular disease, although it is often asymptomatic [7].

Subclavian artery thromboses are typically caused by atherosclerosis. Far less commonly they are due to autoimmune conditions and vasculitides like Takayasu arteritis, fibromuscular dysplasia, congenital causes like a right-sided aortic arch, radiation exposure, coagulopathy, or mechanical obstruction including thoracic outlet syndrome [1,2]. In the appropriate clinical setting, using a cutoff of >10mmHg inter-arm variability in blood pressure has a 99% negative predictive value and a 13% positive predictive value for subclavian artery thrombosis [8]; greater than 15-20mmHg increases this to 67-100% [3,9]. Treatment involves endovascular or surgical revascularization of the affected artery. Untreated arterial thrombosis can lead to critical limb ischemia and compartment syndrome. Cases of acute thrombus occluding the internal mammary artery and causing ischemia or infarction are far less common than those caused by steal syndrome, yet have been documented in the literature [10-12].

Our patient with known extensive coronary atherosclerosis, severe ischemic cardiomyopathy, and atrial fibrillation presented with a significant inter-arm blood pressure differential and ventricular tachycardia, this prompted us to obtain immediate CT angiography of the aorta in order to quickly assess the patient for the surgical emergency of thoracic aortic dissection as well as for any other anatomic abnormalities. It revealed an occlusion of his subclavian artery, which compromised his LIMA and ultimately led to his death via VT. Whether this was a consequence of severe coronary-subclavian steal syndrome or full occlusion of the LIMA graft by thrombus, however, is unclear.

Certainly, multiple conditions predisposed the patient to subclavian artery disease and VT. For example, the patient had a history of radiation to his thyroid bed, neck, and mediastinal nodes, which would have increased his pre-existing risk for subclavian artery atherosclerosis and stenosis given his extensive coronary artery disease [13]. This stenosis, in turn, may have led to coronary-subclavian steal syndrome causing ischemia of the myocardium and resultant VT and would have also predisposed the vessel to thrombosis.

Alternatively, the patient's mid-subclavian artery occlusion may have directly compromised blood flow at the takeoff point of his LIMA. He possessed several risk factors for thrombotic or embolic disease even beyond his suspected atherosclerosis. For example, his old LV thrombus may have embolized at some point between its discovery and eventual resolution on echocardiogram [14], lodging itself in the subclavian artery. He had also developed an increasing burden of atrial arrhythmias since the cessation of anticoagulation for the LV thrombus. His CHA2DS2-VASC score was 4 (for age, heart failure, hypertension, and vascular disease), placing him at a 4.8% risk for CVA and 6.7% risk for cerebrovascular accident, transient ischemic attack, or other embolisms per year [15], including subclavian artery embolism. Finally, we cannot definitively state whether the patient had any hypercoagulable disorders, as his acute presentation and arterial thrombosis necessitated immediate initiation of a heparin infusion which would have interfered with this workup. 

Regardless of the etiology, the patient did present with a subclavian artery occlusion without flow into the LIMA on both CT angiography and invasive coronary angiography. This occlusion then caused either severe coronary-subclavian steal syndrome, as blood flow reversed through the LIMA to attempt to supply the compromised subclavian artery, or the occlusion directly blocked blood flow to the LIMA causing an infarct. For our patient with a poor coronary reserve and a LIMA graft, this occlusion and resultant ischemia proved to be highly significant.

One final note should be made regarding the management of VT in a patient who is actively taking dofetilide. Class III antiarrhythmics, such as dofetilide and amiodarone, can prolong the QT interval and therefore not recommended. Class Ib antiarrhythmics, such as lidocaine, are a safer alternative. Lidocaine holds a weak recommendation from the ACC/AHA/ESC 2006 guidelines supporting its use in ventricular tachycardia thought to be related to ischemia [16].

## Conclusions

Our case illustrates the importance of maintaining a broad differential diagnosis when a patient presents with a significant blood pressure differential between arms. Symptomatic subclavian artery thromboses are rare but can have significant morbidity, particularly in patients with prior coronary artery bypass grafting. Subclavian artery thrombosis should be considered in the differential diagnosis in such patients with significant blood pressure differentials and with signs of new-onset myocardial ischemia.

## References

[1] Mubarik A, Iqbal AM (2020). Subclavian Artery Thrombosis. StatPearls.

[2] Sanders RJ, Hammond SL, Rao NM (2007). Diagnosis of thoracic outlet syndrome. J Vasc Surg.

[3] Lobato EB, Kern KB, Bauder-Heit J, Hughes L, Sulek CA (2001). Incidence of coronary-subclavian steal syndrome in patients undergoing noncardiac surgery. J Cardiothorac Vasc Anesth.

[4] Mehta Rajendra H, Suzuki T, Hagan PG (2002). Predicting death in patients with acute type A aortic dissection. Circulation.

[5] Nienaber CA, Eagle KA (2003). Aortic dissection: new frontiers in diagnosis and management, Part I: from etiology to diagnostic strategies. Circulation.

[6] Saha T, Naqvi SY, Ayah OA, McCormick D, Goldberg S (2017). Subclavian artery disease: diagnosis and therapy. Am J Med.

[7] Gutierrez GR, Mahrer P, Aharonian V, Mansukhani P, Bruss J (2001). Prevalence of subclavian artery stenosis in patients with peripheral vascular disease. Angiology.

[8] English JL, Carell ES, Guidera SA, Tripp HF (2001). Angiographic prevalence and clinical predictors of left subclavian stenosis in patients undergoing diagnostic cardiac catheterization. Catheter Cardiovasc Interv.

[9] Osborn LA, Vernon SM, Reynolds B, Timm TC, Allen K (2002). Screening for subclavian artery stenosis in patients who are candidates for coronary bypass surgery. Catheter Cardiovasc Interv.

[10] Barlis P, Brooks M, Hare DL, Chan RK (2006). Subclavian artery occlusion causing acute myocardial infarction in a patient with a left internal mammary artery graft. Catheter Cardiovasc Interv.

[11] Akgüllü C, Eryılmaz U, Zencir C, Güngör H (2014). Management of a subclavian artery thrombosis causing acute anterior wall infarction and concurrent left arm ischemia in a patient with prior coronary bypass. Turk Kardiyol Dern Ars.

[12] Wu C-H, Sung S-H, Chang JC-Y, Huang C-H, Lu T-M (2009). Subclavian artery thrombosis associated with acute ST-segment elevation myocardial infarction. Ann Thorac Surg.

[13] Sylvester CB, Abe J-I, Patel ZS, Grande-Allen KJ (2018). Radiation-induced cardiovascular disease: mechanisms and importance of linear energy transfer. Front Cardiovasc Med.

[14] Velangi PS, Choo C, Chen K-HA (2019). Long-term embolic outcomes after detection of left ventricular thrombus by late gadolinium enhancement cardiovascular magnetic resonance imaging. Circ Cardiovasc Imaging.

[15] Friberg L, Rosenqvist M, Lip GYH (2012). Evaluation of risk stratification schemes for ischaemic stroke and bleeding in 182 678 patients with atrial fibrillation: the Swedish Atrial Fibrillation cohort study. Eur Heart J.

[16] Zipes DP, Camm AJ, Borggrefe M (2006). ACC/AHA/ESC 2006 guidelines for management of patients with ventricular arrhythmias and the prevention of sudden cardiac death: a report of the American College of Cardiology/American Heart Association Task Force and the European Society of Cardiology Committee for Practice Guidelines (Writing Committee to Develop Guidelines for Management of Patients With Ventricular Arrhythmias and the Prevention of Sudden Cardiac Death). J Am Coll Cardiol.

